# Mitochondrial Morphofunctional Alterations in Smooth Muscle Cells of Aorta in Rats

**DOI:** 10.1155/2014/739526

**Published:** 2014-02-06

**Authors:** María del Carmen Baez, Mariana Tarán, Candelaria Llorens, Ariel Balceda, María de La Paz Scribano, Patricia Pons, Mónica Moya

**Affiliations:** ^1^Instituto de Investigación en Ciencias de la Salud Humana (IICSHUM), Universidad Nacional de La Rioja, La Rioja, Argentina; ^2^Cátedra de Física Biomédica, Facultad de Ciencias Médicas, Universidad Nacional de Córdoba, Córdoba, Argentina; ^3^Becaria Secyt, Universidad Nacional de Córdoba, Córdoba, Argentina; ^4^Cátedra de Física Biomédica, Facultad de Ciencias Médicas, Universidad Nacional de La Rioja, La Rioja, Argentina; ^5^Centro de Microscopía Electrónica, Facultad de Ciencias Médicas, Universidad Nacional de Córdoba, Argentina; ^6^Los Médanos 3155, Alto Verde, 5009 Córdoba, Argentina

## Abstract

In an experimental model of atherogenesis induced by hyperfibrinogenemia (HF), the pharmacological response of vitamin E was studied in order to assess its antioxidant effect on the mitochondrial morphofunctional alterations in aortic smooth muscle cells. Three groups of male rats were used: (Ctr) control, (AI) atherogenesis induced for 120 days, and (AIE) atherogenesis induced for 120 days and treated with vitamin E. HF was induced by adrenalin injection (0.1 mg/day/rat) for 120 days. AIE group was treated with the administration of 3.42 mg/day/rat of vitamin E for 105 days after the first induction. Mitochondria morphology was analyzed by electronic microscopy (EM) and mitochondrial complexes (MC) by spectrophotometry. In group AI the total and mean number of mitochondria reduced significantly, the intermembranous matrix increased, and swelling was observed with respect to Ctr and AIE (*P* < 0.01). These damages were related to a significant decrease in the activity of citrate synthase and complexes I, II, III, and IV in group AI in comparison to Ctr (*P* < 0.001). Similar behavior was presented by group AI compared to AIE (*P* < 0.001). These results show that vitamin E produces a significative regression of inflammatory and oxidative stress process and it resolved the morphofunctional mitochondrial alterations in this experimental model of atherogenic disease.

## 1. Introduction

There is great interest in the prevention of atherogenic disease with a focus on the effectiveness of pharmacological agents, among them, the antioxidants for primary prevention [1, 2]. The evolution of physiopathological knowledge of atherogenesis has given the inflammatory process and oxidative component an importance equivalent to the lipid accumulation in the vascular wall [3, 4]. Cardiovascular risk factors such as hyperfibrinogenemia generate oxidative stress; their toxic effects on biomolecules at the level of vascular layers, associated with the metabolic degradation and redox-sensitive signaling, induce the development of the pathology [5]. The dysfunctional endothelium loses its ability to regulate its vital functions, acquiring procoagulant properties instead of anticoagulant, and reduces the bioavailability of nitric oxide (NO) by oxidative inactivation due to the excessive production of superoxide and hydrogen peroxide in the vascular wall. These changes constitute the most characteristic and the earliest systemic phenomena of the endothelial dysfunction, *sine qua non* condition for atherogenesis [6, 7]. As we have demonstrated in previous works [8, 9], the endothelial activation induced by hyperfibrinogenemia (HF) would lead to the noncontrolled synthesis of reactive oxygen species (ROS) and they are dominant signaling mediators for the basis of the vascular inflammation in atherogenesis. The aggression that these ROS impose on biomembranes destabilizes the structure and function of the cell and its organelles [10, 11].

On the other hand, the effects would act at mitochondrial level modifying the efficacy of electron transport chain, the oxygen concentration, availability of electron donors, cytokine production, and the activity of antioxidant defenses [12, 13]. As a result of the mitochondrial respiratory inhibition, electrons are accumulated which causes that NADH-ubiquinone reductase (complex I) and the ubiquinol cytochrome c reductase (complex III), that possess ubiquitin as common component, increase superoxide generation. For this, peroxynitrite and hydrogen peroxide perpetuate the pathognomonic lesions of atherogenesis [14]. This excess in the formation of highly reactive molecules, such as ROS and reactive nitrogen species, saturates the natural antioxidant defense mechanisms that are in charge of neutralizing their toxicity and avoid that their concentrations become pathological [15, 16].

There are exogenous antioxidant drugs such as alpha-tocopherol (vitamin E), a liposoluble vitamin, and the main antioxidant in cellular membranes and in low density lipoproteins (LDL). Its importance lies in the presence of a –OH group in its structure (alpha-tocopherol-OH) with a hydrogen (H) that is easily separated from the molecule [17, 18].

The purpose of this investigation was to study, in an experimental model of atherogenesis induced by HF, the antioxidant effect of vitamin E on the mitochondrial morphofunctional alterations in aortic smooth muscle cells in order to develop therapeutic strategies to prevent this pathology of high impact and global prevalence.

## 2. Materials and Methods

Male Wistar rats of 250 ± 20 g average weight were used. Animals were bred and housed under controlled conditions, maintained at room temperature (20°C ± 2°C), and fed with a balanced Cargill's diet. The investigation was carried out according to the Guide for the Care and Use of Laboratory Animals published by the US National Institute of Health (NIH publication number 85–23, revised 1996). The ethical commission of the School of Medicine of the National University of Cordoba has also approved the experimental animal procedures.

A total of 36 rats were used and each group had 12 animals which were sequentially studied and classified into the following experimental situations

- group Ctr: control (without hyperfibrinogenemia induction);
- group AI: hyperfibrinogenemia induced for 120 days;
- group AIE: hyperfibrinogenemia induced for 120 days + treatment with vitamin E for 105 days.

No deaths were registered and there were no animals excluded in any of the groups studied.

HF induction was made with subcutaneous injections of adrenaline (0.1 mg/day/rat) for 120 days [9, 19]. Pharmacological treatment was carried out with oral administration of vitamin E diluted in double distilled water, in doses of 3.42 mg/day/rat (1 UI = 0.45 mg), equivalent to 800 mg indicated in humans [18]. It began on day 15 after the first HF induction and for a period of 105 consecutive days with a 1 mL syringe adapted with a catheter that allowed the deposit of the adequate amount on the esophagus to avoid regurgitation by the animal. Material for the electron microscopy was obtained by “*in toto*” slices with 2 mm rings of thoracic aorta in all the studied groups. The tissue was fixed in Karnovsky's fixative (1965) [20] made up of a mix of formaldehyde 4% and glutaraldehyde 1.5% in the cacodylate buffer 0.1 M for a period of at least 2 hours at room temperature. The ultrathin sections obtained in a Jeol Jum-7 ultramicrotome with diamond knife were mounted in nickel grids and coloured with uranyl acetate in alcoholic solution and lead citrate, and they were observed and photographed in a Leo 906E (Carl Zeiss, Jena, Germany) electron microscope.

In order to assess the morphological characteristics of mitochondria, measurements were made in a total of 8 photos per group (*n* = 8) at random and all presented 27800X. The reference area used was 1986 *μ*m^2^ for their morphometric analysis and there was a 3-grade classification according to the percentage of mitochondrial modifications observed:

 grade 1: normal appearance with a size of up to 1.7 *μ*m;
 grade 2: normal size with visible crests, but unusual;
 grade 3: their size corresponds to 50% of normal size. Very dilated mitochondria with high disorganization of internal and external membranes in some zones, with visible and dilated crests. Clear matrix with electron dense remains close to the outer membrane and presence of vacuoles.

For the mitochondrial isolation was used a method based on the extraction of all the thoracic aortas (approximately 200 mg each) from rats of different groups and they were washed and suspended in a cold isolation buffer and they were immediately homogenized. Later, they were centrifuged and resuspended until the mitochondrial pellet was obtained, which was resuspended in the isolation buffer (tissue/buffer ratio: 1/1) [21]. Protein concentration was measured with Bradford method [22]. This mitochondrial pellet helped to determine the functionality of Krebs cycle through the determination of the citrate synthase activity [21]. Spectrophotometric procedures were carried out in order to quantify the enzymatic activity of the complexes I to IV of the respiratory chain: CI: NADH-ubiquinone reductase; CII: succinate-ubiquinone reductase; CIII: ubiquinone-cytochrome c reductase; CIV: cytochrome c oxidase, according to the techniques of Vyatkina, Trounce, and Jarreta [21, 23, 24].

### 2.1. Statistical Analysis

For the analysis of the results of the independent variables (animal groups studied) with respect to the complexes, InfoStat (InfoStat version 2008, Group InfoStat, Argentina) was used; the normality and homogeneity tests were carried out with Shapiro-Wilk test and analyzed with MANOVA; Hotellings test was used for post-testing. For the mitochondrial morphologies analysis, the program AxioVision 4.8 (Copyright © 2009 by Carl Zeiss, Jena) was used to evaluate the mitochondrial structure and it was analyzed with Fisher test for qualitative variables. A significance level of *P* < 0.05 was established for all cases.

## 3. Results

The results of the mitochondrial quantification of the studied groups are shown in Table 1.

**Figure 1:**
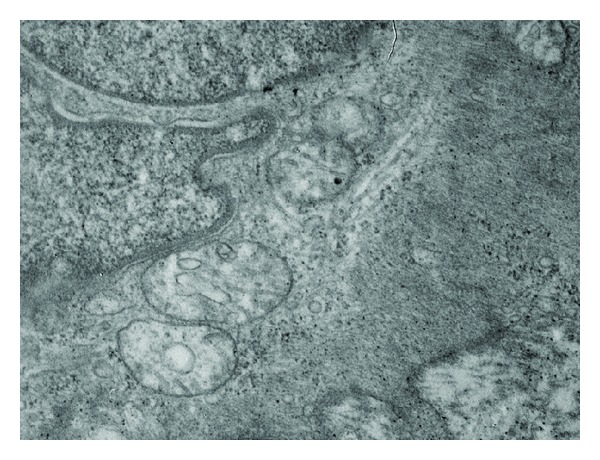
Microphotograph of mitochondria of the group with atherogenesis induced for 120 days AI shows mitochondria with disorganized crests, enlarged with clear matrix and variable sizes. They are dispersed and associated with several vesicles (⇑).

**Figure 2:**
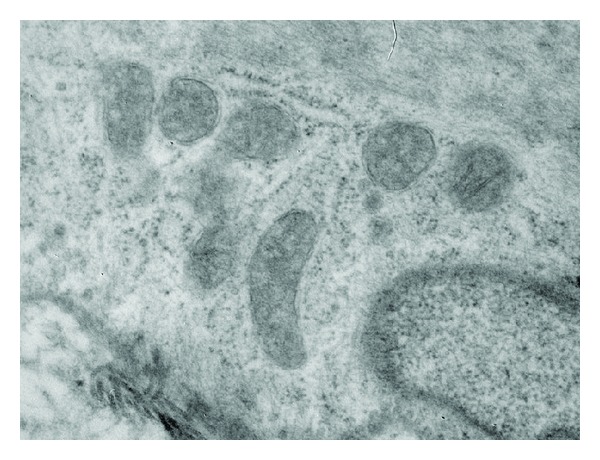
Microphotograph of mitochondria of group Ctr where we can observe mitochondria without changes in the structure of membranes and crests, maintaining normal form and size (⇑).

The total and mean number of mitochondria decreased significantly in the group AI (Figure 1) with respect to control group (Figure 2) (*P* < 0.01). At the same time, it was observed that mitochondria in the group AI presented a significant size increase and clearing of the matrix with disorganized crests associated with several vesicles, changes compatible with mitochondrial swelling.

When alteration grade 3 was compared, it showed a significant difference between group AI (67.31%) and group Ctr (0.01%) (*P* < 0.001).

**Figure 3:**
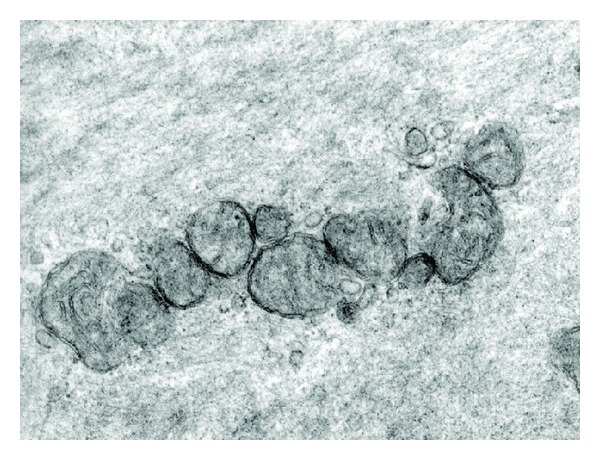
Microphotograph of mitochondria of group AIE with atherogenesis induced for 120 days and treated with vit. E where we can see mitochondria of normal characteristics with crest recovery (⇑).

In the group AIE (Figure 3), mitochondria with normal characteristics and crest recovery were observed; injury regression manifested by involution of alteration grade 3 to 18% and significantly set back to alteration grade 1 (62%) with respect to the group AI.

The results of the enzymatic activity of citrate synthase (*μ*mol CoA/min/mg protein) are shown in Table 2.

It was observed that, as HF persisted, the citrate synthase activity decreased significantly as it is reflected in the group AI and there was a significant difference in relation to group Ctr (*P* < 0.001). In the group AIE a significant increase in the activity of this enzyme was observed in comparison to AI (*P* < 0.001), but without normalizing its activity in Ctr (*P* < 0.01).

The results of the enzymatic activity of the mitochondrial respiratory chain complexes are shown in Table 2.

The activity of complex I (*μ*mol NADH/min/mg protein) in the mitochondrial respiratory chain decreased significantly in group AI in relation to control group Ctr (*P* < 0.001). The administration of vitamin E for 105 days in group AIE normalized the activity of complex I and increased significantly in relation to animals from group AI (*P* < 0.001).

When the results of complex II (fmol succinate/min/mg protein) were analyzed, a marked decrease in the group AI was verified in relation to control group Ctr (*P* < 0.001). When the activity of the said complex was determined in the group AIE, it showed an important increase in its enzymatic activity in relation to group AI (*P* < 0.001).

Also, a decrease in the enzymatic activity of complex III (*μ*mol ubiquinone/min/mg protein) of the mitochondrial respiratory chain was proved in AI in relation to control Ctr (*P* < 0.001). When the activity in the group AIE was analyzed, there was an important increase in the activity in comparison to group AI (*P* < 0.001), but it remained significantly reduced with respect to control Ctr (*P* < 0.01).

The enzymatic activity of complex IV (*μ*mol iron-cytochrome c/min/mg protein) decreased significantly in the group AI in relation to control Ctr (*P* < 0.001). When animals were treated with vitamin E for 105 days in AIE, the enzymatic activity of this mitochondrial complex increased significantly in relation to group AI (*P* < 0.001) and even in relation to group Ctr (*P* < 0.01).

## 4. Discussion

In a previous work we demonstrated that the HF state reflected the inflammatory process and the increase in oxidative stress in the vascular wall, altering the vasodilator reaction with changes in the vascular flow and ischemia as a consequence of the endothelial dysfunction [8, 9]. These mechanisms affect the mitochondrial function and morphology as we observed in the electron microscopy (Figure 1). The free radicals that formed in excess during oxidative stress triggered by hyperfibrinogenemia would generate lipid peroxidation in the mitochondrial membranes causing changes in their morphology and integrity and, therefore, the dysfunctional selective permeability allowed the massive entry of ions and water changing their density, area, and normal size [25]. Other authors that studied cardiac contractility under ischemic conditions have shown that the protective ability of mitoK_ATP_ openers is associated with preservation of the mitochondrial intermembrane spaces volume, leading to the preservation of adenine nucleotide compartmentation and energy transfer [26]. When mitochondrial membrane potential was perturbed, it will cause changes in matrix and mitochondrial intermembrane space volume [27], consequently, K^+^ diffusion into the matrix will decrease, the mitochondrial matrix will contract, and the mitochondrial intermembrane space will expand, maybe in the mitochondrial of smooth muscle cells of vascular layers, has occurred the same alteration in the activity of mitoK_ATP_, but this point should be better studied in this tissue [28]. The morphological alterations were probably due to the temporary transition pore opening during ischemia that affects the ion homeostasis at cell and mitochondrion level. This leads to the swelling of the matrix and subsequent breaking of the outer mitochondrial membrane; this endogenous mitochondrial oxidative stress generated an increase in the proatherogenic risk [29, 30]. After the turbid swelling there was a disappearance of mitochondrial crests called crystolysis, an injury that is reversible in its initial stage when vitamin E has been administered, as it is observed in our results (Figure 3, Table 1). Probably the inhibition of proinflammatory cytokines, caused by alpha-tocopherol, would generate a decrease of reactive nitrogen species and its impact on mitochondrial morphology as shown in the group treated [31]. In this way, it prevented mitochondrial dysfunction and the subsequent oxidative and nitrosative stress, perhaps reestablished the balance between fusion/fission, and would preserve the production of cellular energy (ATP) [32].

The mitochondrial fission is regulated by proteins located on the outer membrane of the mitochondrion. Antioxidants such as vitamin E can prevent the damage of mitochondrial proteins, avoiding a decrease in the activity of the manganese superoxide dismutase which is crucial for the pathogenesis of atherosclerosis [29, 32].

The mitochondrial morphological alterations are associated with modifications in the functionality of the respiratory chain, showing that through oxidative stress HF could have impact on mitochondrial morphofunctionality [33]. These oxidants irreversibly inhibit the components of the mitochondrial respiratory chain through the enzymatic inactivation of dehydrogenated NADH (complex I) and dehydrogenated succinate (complex II), as well as inhibiting ATP synthase [13, 33]. As a free radical, NO reacts with heme iron to form a nitrosyl inclusion complex and, when it interacts with the prosthetic heme groups of cytochromes, it produces the interruption of oxidative phosphorylation, altering the cellular metabolic activity [25, 34]. The lipids affected in the mitochondrial membranes entail a functional alteration of the mitochondrial respiratory chain since complexes I and III, sources of oxygen reactive species, modify their activity as well as complex II (this one probably due to lack of succinate coming from the Krebs cycle). The modification of complex IV would be a consequence of the scarcity of electrons after the oxidative damage present in atherogenesis [35].

The administration of vitamin E for long periods increases the activity of the Krebs cycle, shown by the growth in the enzymatic activity of citrate synthase, increasing the substrate for the mitochondrial complexes I, II, and IV. The results allow us to infer that the recommended administration of vitamin E should be for a period of 105 days in order to accomplish a reversion of the alterations in the mitochondrial morphofunctionality.

Since complexes I and II are the electron sources for the normal operation of the respiratory chain, the normalization of their activity is translated into a global improvement in the activity of the respiratory chain, as it is observed in the treatment with vitamin E [36].

The action mechanism of vitamin E to neutralize free radicals would be formed by an innocuous product, alpha-tocopherol-O. The alpha-tocopherol-O's behaviour is to be less reactive, stopping the chain reaction of free radicals due to its inability to attack the lateral chains of adjacent fatty acids [15]. Phospholipids of the mitochondrial membranes have an affinity for alpha-tocopherol; therefore, it is very concentrated in these places. Other functional aspects of vitamin E include the inhibition of platelet aggregation, inhibition of the proliferation of smooth muscle, and the neutralization of peroxynitrites, while NO availability increases [16]. Vitamin E would inhibit the lipid peroxidation that takes place in the polyunsaturated fatty acids of the lipids and it is started by the abstraction of a hydrogen atom from methyl groups by strong oxidant species such as superoxide anion [36]. This event inactivates the enzymes integrated in the biological membranes and decreases the fluidity of the membrane. Therefore, vitamin E would improve the mitochondrial function since all the complexes of the respiratory chain, except for complex II, depend on the integrity of phospholipids as to structure and function, allowing the adequate electron transport to complex IV [14, 36] as it is corroborated in the batch treated for 105 days with vitamin E (Table 2).

These results show that vitamin E produces a significative regression of oxidative stress process and it resolved the morphofunctional mitochondrial alterations in this experimental model of atherogenic disease, but the experimental results cannot always be translated into clinical situations.

## Figures and Tables

**Table 1:** Mitochondrial quantifications in the smooth muscle of the thoracic aorta in rats with atherogenesis induced by hyperfibrinogenemia and treated with vitamin E.

Measurements	Ctr	AI	AIE
Total number of mitochondria*	54	34	46
Mean number of mitochondria*	9 ± 0.30	7 ± 0.46	6.9 ± 0.48
Mean area of mitochondria (*μ*m^2^)	465.61 ± 28.06	552 ± 65.73	592.29 ± 16.28
Alteration grade (%)			
Grade 1	87. 24	3.5	62
Grade 2	12.75	39.78	20
Grade 3	0.01	67.31	18

Ctr: control; AI: atherogenesis induced for 120 days; AIE: atherogenesis induced for 120 days and treated with vit. E (*n* = 12).

*Total area measured 1986 *μ*m^2^.

**Table 2:** Enzymatic activity of citrate synthase and of the complexes of the respiratory chain (I to IV) in the thoracic aorta of rats with atherogenesis induced by HF and treated with vitamin E.

	Ctr	AI	AIE
Citrate synthase (*μ*mol Co.A/min/mg protein)	0.3596 ± 0.004	0.080 ± 0.001	0.31 ± 0.012
Complex I (*μ*mol NADH/min/mg protein)	0.0646 ± 0.00131	0.0083 ± 0.003	0.07 ± 0.0025
Complex II (fmol succinate/min/mg protein)	0.0566 ± 0.00184	0.0406 ± 0.00174	0.0676 ± 0.00195
Complex III (*μ*mol ubiquinone/min/mg protein)	0.2617 ± 0.010	0.07 ± 0.0132	0.2094 ± 0.01
Complex IV (*μ*mol iron-cytochrome c/min/mg protein)	0.1712 ± 0.0017	0.031 ± 0.005	0.28 ± 0.01

Ctr: control; AI: atherogenesis induced for 120 days; AIE, atherogenesis induced for 120 days and treated with vit. E (*n* = 12).

Activities of citrate synthase and complexes I–IV. Data are means ± SEM: AI versus Ctr: *P* < 0.001; Ctr versus AIE: *P* < 0.01; AIE versus AI: *P* < 0.001.
